# Minimally Invasive Harvesting Technique for Costal Cartilage Graft: Donor Site, Morbidity and Aesthetic Outcomes

**DOI:** 10.3390/jcm12103424

**Published:** 2023-05-12

**Authors:** Umberto Committeri, Antonio Arena, Emanuele Carraturo, Simona Barone, Giovanni Salzano, Domenico Mariniello, Giacomo De Riu, Luigi Angelo Vaira, Francesco Giovacchini, Luigi Califano, Pasquale Piombino

**Affiliations:** 1 Maxillofacial Surgery Unit, Department of Neurosciences, Reproductive and Odontostomatological Sciences, University of Naples “Federico II”, 80131 Naples, Italy; 2 Plastic, Reconstructive, Aesthetic Surgery Unit, Department of Public Health, Federico II University, 80138 Naples, Italy; 3 Maxillofacial Surgery Operative Unit, University Hospital of Sassari, 07041 Sassari, Italy; 4 Maxillofacial Surgery Unit, Santa Maria Della Misericordia Hospital, 06129 Perugia, Italy

**Keywords:** cartilage graft, costal cartilage, rib cartilage, nasal reconstruction, Vancouver Scar Scale, nose

## Abstract

Cartilage grafts are well-known as being reliable in reconstructive surgery for craniofacial pathologies. The aim of this study is to describe a new technique which requires an incision smaller than 1.5 cm but is still effective for harvesting cartilage graft. Thirty-six patients who underwent costal cartilage harvesting for septorhinoplasty have been included in this study, admitted from January 2018 to December 2021. Out of 36 patients, 34 have not reported any major complications, and two cases were followed up for pneumothorax. There were no infections and no chest wall deformities. All patients reported minimal pain at the donor site. The Vancouver Scar Scale was used to evaluate the entity of the postoperative scarring phenomena. This scale total ranges from 0 (representing normal skin) to a maximum score of 13 (representing worst scar imaginable). The results were 1.53 SD ± 0.64 (on average) 1 week after the surgical procedure and 1.28 SD ± 0.45 (on average) at the 6 months follow-up. This minimally invasive method provided a valid and effective surgical technique for cartilage graft. Despite the limitations of the case series, it seems that this procedure might be comparable to other and well-established traditional procedures and could be even preferred when the minimal invasiveness is mandatory.

## 1. Introduction

Autogenous cartilage grafts are still considered the standard of care in patients undergoing augmentation of the nasal dorsum [1]. This harvesting technique has been and still is useful to manage a large quantity of defects and reconstructive needs. The majority of the minor deformities are usually corrected using conchal or septal cartilage or relying on diced cartilage wrapped in Surgicel (SURGICEL^®^ Absorbable Hemostat, Ethicon US, LLC jnjmedicaldevices) or fascia [2]. When a considerable amount of material is needed for rhinoplasty and nasal reconstruction, rib cartilage is regarded as a versatile and appealing option. Costal cartilage grafts have a wide range of uses in various surgical and reconstructive procedures. Here are a few examples:

Rhinoplasty: Costal cartilage grafts are commonly used in rhinoplasty surgery to provide structural support and help reshape the nose. They can be used to augment the nasal tip, bridge or sidewalls, as well as to correct a deviated septum [3];

Ear reconstruction: Costal cartilage grafts are also frequently used in ear reconstruction surgery to create a new ear or to repair a damaged or missing ear. The cartilage can be shaped and sculpted to match the patient’s other ear and to create a natural-looking result [4];

Cleft palate repair: Costal cartilage grafts can be used to repair cleft palate defects, which occur when the roof of the mouth doesn’t fully develop. The grafts can help create the necessary support and structure for the palate and can be used in combination with other tissue or bone grafts [5];

Breast reconstruction: Costal cartilage grafts can also be used in breast reconstruction surgery to help create a natural-looking breast mound. The cartilage can be used to provide additional support and contour to the breast tissue [6];

Facial reconstruction: Costal cartilage grafts can be used in facial reconstruction surgery to help repair facial defects resulting from trauma, cancer surgery or congenital anomalies. The cartilage can be used to provide support and structure to the affected area and can be shaped and sized to fit the specific needs of the patient [7,8];

Laryngotracheal reconstruction: Costal cartilage grafts are used widely in the laryngotracheal reconstruction in patients (especially pediatrics) affected by laryngotracheal stenosis [9].

A great number of advantages render the use of rib cartilage strongly preferred in septorhinoplasty and nasal reconstruction [10]. First of all, rib cartilage has intrinsic qualities such as its strength and abundance. Therefore, its availability, biocompatibility and ease of use make it a popular choice in aesthetic and reconstructive surgery [11]. However, if the costal cartilage graft is considered the best option, the conventional surgical technique for harvesting is still burdened by doubts on its viability. With traditional procedures, a great amount of large, full-thickness cartilage pieces is harvested from the chest wall and cut, shaped and remolded to create the desired form. A large quantity of cartilage is made unusable for future use by the procedure itself. These traditional procedures for costal cartilage harvesting come with numerous problems such as pain, deformity of the thoracic bones, synchondrosis and a large scar. It is also true that this procedure is burdened by great pain at the donor site [12]. Despite the well-known drawbacks of this surgical technique, there is ongoing debate in literature. Bone grafts are also described in literature, mainly in combination with cartilage graft to ensure reconstruction of great deformity and represent a more difficult method with a higher complication rate, reserved only for selected cases [5,6,7,8,9,10,11,12,13]. The aim of this study is to illustrate a minimally invasive technique, effective at harvesting costal cartilage and useful in guaranteeing minimally unaesthetic surgical scars.

## 2. Case Series

For this study a cohort of 36 patients was selected who underwent costal cartilage harvesting for septorhinoplasty, admitted from January 2018 to December 2021 in the Department of Maxillofacial Surgery of “Federico II” University, Naples. The cohort was composed of 16 females and 20 males, and the average age was 25.75 (SD ± 6.03). Among them were 14 patients suffering from cleft lip nasal deformity, 14 from a severely traumatic deviated nose, 5 from saddle nose and 7 needing a secondary septorhinolasty after a failing cosmetic primary rhinoplasty. In this study, patients requiring costal cartilage graft for augmentation rhinoplasty were included depending on the underlying pathology:

1. Patients suffering from congenital nasal malformations, who needed a costal cartilage graft in order to restore dorsal height and/or severe alar collapse, as in cleft lip and palate.
2. Patients suffering from saddle nose, broad nose; this is not pathological in strict sense, but as typical traits in some ethnic groups (e.g., Mongolic/African people) which therefore needed extra cartilage graft in order to restore dorsal height/tip projection.
3. Patients suffering from serious nasal trauma who needed accurate reconstruction.
4. Patients needing secondary (revision) septorhinoplasty who had a previous failed rhinoplasty (Table 1).

All of these patients were included as conchal/auricolar or septal cartilage graft was not indicated nor enough to cover their tissue deficiency.

Patients presenting with psychiatric conditions, or already enrolled in other studies, were excluded, as well as patients who abandoned the follow-up period (under 3 months), patients not adequately data- and/or photo-recording and eventually patients who had unrealistic and unsatisfactory expectations. Participants provided written consent. Patients were evaluated by using the Vancouver Scar Scale (Baryza MJ and Baryza GA, 1995) [14] (Table 2).

This study obtained ethical approval from the Human Research Ethics Committee of the University Hospital Federico II of Naples, Italy (protocol number 88/20). Preoperatively, all patients underwent computed tomographic examinations to study the rate of possible cartilage calcification.

### 2.1. Surgical Planning

Radiological protocol consisted in a high-resolution computed tomography (CT) scan of the thorax with an axial slice thickness of 1 mm. The DICOM datasets were segmented into 3-dimensional virtual bone models of skeleton in order to evaluate skeletal maturity and the presence of cartilage with the software InVesalius, (InVesalius 3.0, by CTI, Centro de Tecnologia da Informação Renato Archer).

The ribs generally considered in this phase include the 5th, 6th and 7th ones, due to their position, depth and length. Typically, the seventh rib is preferred for the graft.

### 2.2. Surgical Procedure

During the procedure, the patient was under general anesthesia, and the position and shape of the donor-site costal cartilage was checked by palpation.

**Figure 1:**
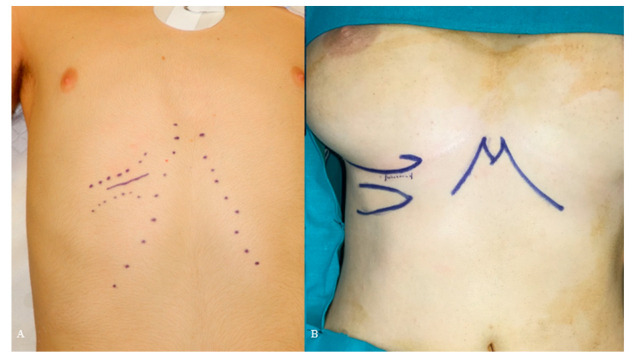
Skin incision in male (**A**) and female (**B**) patient.

A 25-gauge percutaneous needle was used before the incision to identify and evaluate the correct position and the borders of the available cartilage, in order to minimize the risks of useless harvesting. In male patients, the incision is usually placed directly over the chosen rib to facilitate the dissection (Figure 1A). In female patients, the skin incision is marked approximately 5 mm above the inframammary fold (Figure 1B).

**Figure 2:**
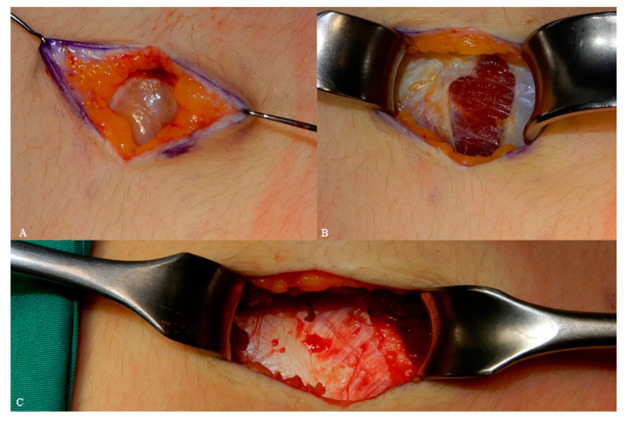
Tissue incision, skin and fat (**A**), fascia (**B**) muscle (**C**).

The incision is made slightly medially to the costochondral junction. A deep skin incision was made through cutis and subcutaneous fat, exposing the fascia. Adequate surgical view was achieved by means of retractors, and a longitudinal incision was placed between the external oblique muscle and rectus abdominus muscle (Figure 2A–C).

**Figure 3:**
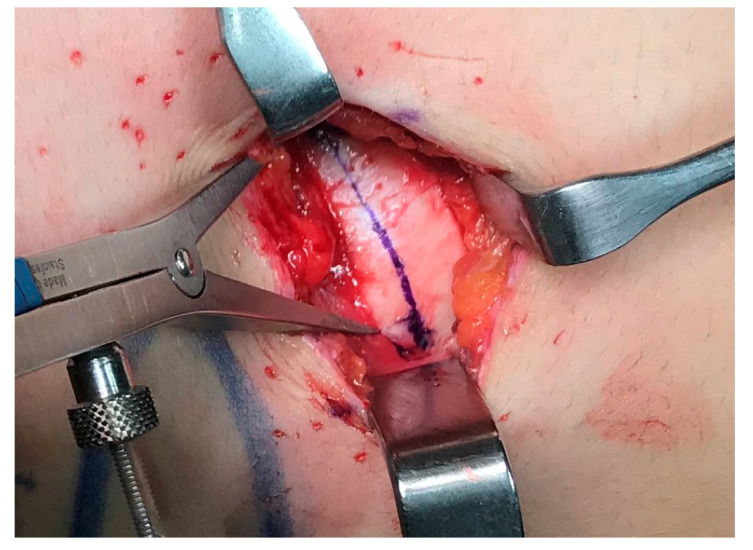
Tissue incision.

The muscles were then dissected in a way parallel to their own fibers. The costal cartilage appeared beneath a layer of loose areolar tissue. The cartilage was exposed and dissected from the perichondrium which has been incised considering the correct length of the graft (3 cm in average, SD ± 0.99) (Figure 3).

**Figure 4:**
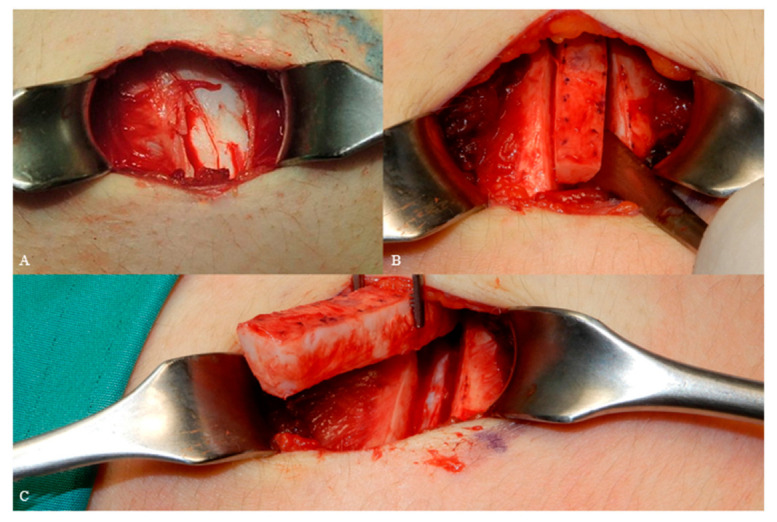
Cartilage exposing (**A**), incision (**B**), harvest (**C**).

After having marked the needed shape and quantity, the cartilage was directly incised with a scalpel number 10 and harvested on the deep plane by means of a rounded, smooth and thin periosteal elevator Figure 4A,B.

Split costa grafts of 1.01 ± 0.15 V (height) × 3 ± 0.99 (length) × 0.31 ± 0.07 (thickness) cm (on average) were harvested, leaving the deep perichondrium and the remaining costal cartilage intact. (Figure 4C).

Then the lateral and inferior aspects of the cartilage were left, and the perichondrium was saturated on the gap left by the graft. Layered suture of muscles and cutaneous layers followed.

A “leak test”, to show that there was no leakage in the thoracic wall, was performed as per routine with the collaboration of the anesthesiologist. The rectus abdominis muscle and external abdominal oblique muscle were lifted with retractors or hooks and saline was injected into the cavity formed and the increasing thoracic pressure by 20 to 30 cm H_2_O via anesthesiologist.

**Figure 5:**
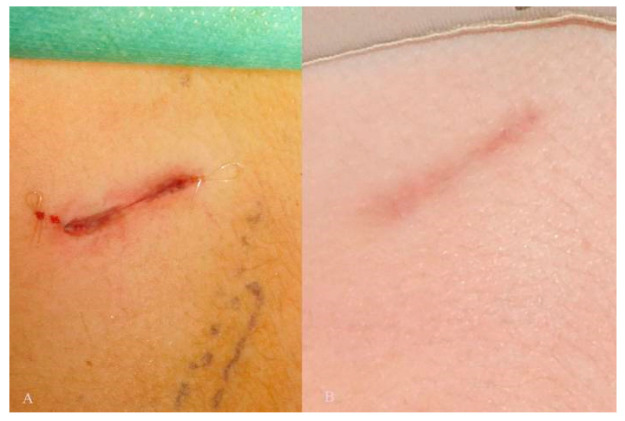
Post-operative suture (**A**), 6 months follow-up (**B**).

The sutures were made with Vicryl 4-0. The skin was closed in non-resorbable material by means of subcuticular suture (Figure 5A,B).

In order to avoid the formation of seroma, a drainage was applied and was then removed in 2–3 days. Management of post-surgical pain was performed by injection of local anesthetic at the donor site (10 cc of 0.75% Ropivacaine). Patients had to lay supine (i.e., lay in bed) for at least 48 h.

### 2.3. Follow-Up

The follow-up period included a postoperative outpatient clinic review at 1 week for suture removal, and then 1 month, 3 months and 6 months after surgery; the general condition of each patient and the scar of the donor site were thoroughly evaluated. Scar conditions were assessed with the Vancouver Scar Scale (Sullivan et al., 1990).

The Scale is one of the standardized appliances for scar wound assessment. It was described by Sullivan in 1990 and is the most used scar assessment method in the world.

It evaluates 4 factors: vascularity, pliability, pigmentation and height of the scar. For pliability the score ranges from 0 to 5, for height and vascularity parameter the score range is 0 to 3 and for pigmentation the range is 0 to 2; therefore, each parameter contains ranked subscales that may be summed to obtain a total score ranging from 0 (representing normal skin) to a maximum score of 13 (representing worst scar imaginable). This questionnaire was given by the surgeon to the single patient and filled at every postoperative clinic visit. The results were 1.53 SD ± 0.64 (on average) 1 week after the surgical procedure and 1.28 SD ± 0.45 (on average) at the 6 months follow-up.

Out of 36 patients, 35 did not report any major and or minor complications both short- and long-term. There was no infection nor chest wall deformities or seroma. One patient presented postoperative pneumothorax. All patients reported minimal pain at the donor site, which was prevented with standard protocol for analgesia.

The results of Vancouver Scar Scale are shown in Table 3.

The majority of patients noticed a difference in scar color, thickness and stiffness, which diminished over time. Most of the patients were also particularly satisfied with the aspect of their scar.

## 3. Discussion

Using cartilage graft for reconstructive and cosmetic purpose in nasal (as well as ear) surgery is not only a growing trend in the field of reconstructive surgery, but sometimes necessary in order to get the best result, where the use of septal cartilage is not possible for various reasons (e.g., absent, damaged or insufficient). Peer et al. were the first to describe the use of cartilage graft in reconstructive surgery. [15]. Since then, many authors have reported technical refinements of the original procedure [16,17,18,19,20,21,22]. Traditionally, the surgical procedure is represented by an extended incision of up to 10 cm, due to the necessity of harvesting the entire costal cartilage segment, as in auricular reconstruction. For this reason, residual scarring is one of the major patients’ cosmetic concerns [22,23,24,25]. This incision approach represents an alternative way to harvest the cartilage in a manner which avoids aesthetic problems, particularly in female patients. Rohrich et al. described an incision shorter than 3 cm while in 2006 Kawanabe et al. suggested a surgical incision of less than 2 cm [13,17,24]. In literature, in these procedures, different surgical complications were reported, such as considerable pain, deformity of the thorax and a long scar at the donor site [21,25,26,27,28]. In young patients, large defects of the chest wall cartilage often led to deformity and other serious complications due to a continuous negative respiratory pressure that can exacerbate deformity, especially in small children. In 1997, Ohara et al. reported four cases of thoracic scoliosis and one case of kyphosis [28]. Even if complications reported in literature are mostly circumscribed to ear reconstruction surgery, a consistent number has also been described for nasal surgery requiring a cartilage graft. The modifications and refinements proposed in this study in order to reduce intraoperative and postoperative complications are as follows: first, the perichondrium is left completely intact at the donor site in order to avoid chest wall deformity and facilitate the cartilage regenerative spontaneous turnover.

Secondly, the entire costal cartilage segment is not harvested, unlike in the traditional method, which involves the use of the entire piece of harvested cartilage graft.

A similar method has been previously described by Michael Lee et al. in 2016. Unlike the technique described in this paper, they harvested the cartilage in a triangular shape [18]. It is preferrable to harvest the pieces in a linear shape, since it is easier to collect even for non-experienced surgeons. Moreover, as is consistent with Takatoshi Yotsuyanagi et al., this method requires a small incision of less than 1.5 cm, with minimal residual scarring and strategic positioning in female patients in the mammary sulcus [17]. Furthermore, in order to avoid any chance of postoperative scars becoming particularly visible, the incision should not extend beyond the medial extent of the inframammary fold. In male patients the scar is not as important as in the female ones because of the great quantity of chest hair that can mask it.

The linear skin incision design alongside with the parcellar harvesting of the cartilage blocks appears to prevent the main complications reported in literature for extensive harvesting. It is evident how harvesting a great quantity of cartilage can be a good opportunity for reconstruction but also can lead to a chest deformity especially in young patients. Small pieces of cartilage 1cm (height) × 3 cm (length) × 0.3 (thickness) cm are harvested.

This amount of cartilage is enough to guarantee at least 3–4 blocks of cartilage are viable to achieve a total nasal reconstruction. The grafts primarily harvested include the spreader graft, alar graft, tip graft, rim graft and columellar graft. If other pieces are required intraoperatively (i.e., dorsal graft or diced graft), an extra 1 × 1 cm piece remnant from the main graft, would be enough for the procedure. The use of this new method results in a lower recurrence of chest wall deformity and residual and disfiguring scarring. There were no infections in the presented series. Two cases of pneumothorax were observed. One case of pneumothorax was observed in a patient with previous chest trauma that has not allowed a proper smooth subperiosteal dissection due to the fibrosis. The patient underwent the insertion of a pleural drainage by the thoracic surgeons and was discharged after 20 days with no sequalae.

In literature, and as described by a meta-analysis by Chen et al. (2023), the most common post-operative complication was determined by warping followed by revision rate, hypertrophic chest scar, contour irregularity, infection, resorption and pneumothorax. Pneumothorax, as the author suggests, had an important asymmetry in the funnel plots of the meta-analysis, suggesting that they were subject to publication bias, most likely secondary to the individual surgical patients (i.e., post-traumatic sequelae) [28,29].

All the patients reported minimal pain at the donor site, which was treated with oral medication. This allowed the authors to promptly and safely discharge the patients on the same day of the procedure.

This study suffers from a series of limitations. First of all, as has been stably adopted this surgical procedure, it has been not possible to provide a full-bodied control group made up by patients undergoing the traditional procedure. Secondly, the long term effects could not be studied due to a lack of adherence to the programmed post-operative checks by a conspicuous number of patients. This only permitted a short follow-up (6 months). At last, the study focused on a single distinct parameter (*The Vancouver Scale Scale*).

## 4. Conclusions

This method allows the minimization of postsurgical biological costs for patients, while obtaining good quality and quantity of cartilage graft for nasal reconstruction in complex cases. This approach would be of help for other clinicians, despite the fact that a larger cohort would be required, as well as longer follow-ups, to better define the technique. It is important to underline how this method in a not-so distant future could be considered obsolete, as bioregenerative medicine and surgery evolves. For instance, acellular dermal matrix (ADM) is a bioengineering technique used nowadays for numerous applications, as Gierek et al. states; these applications include breast surgery; skin surgery; hernia repair; and even dermal injections. Literature still does not describe any use of ADMs in reconstructive surgeries of nasal and ear cartilage; nonetheless, it is a good sign that these surgeries could represent a new field of application for ADMs [30,31].

## Figures and Tables

**Table 1:** Demographic data and diagnoses of the patients.

Patient	Age (Years)	Gender	Diagnosis
**1**	18	M	Cleft lip nasal deformity
**2**	31	M	Severely traumatic deviated nose
**3**	26	M	Severely traumatic deviated nose
**4**	23	M	Cleft lip nasal deformity
**5**	37	F	Cleft lip nasal deformity
**6**	28	M	Secondary to a failing primary cosmetic rhinoplasty
**7**	20	F	Cleft lip nasal deformity
**8**	21	F	Secondary to a failing primary cosmetic rhinoplasty
**9**	31	F	Saddle nose
**10**	22	M	Secondary to a failing primary cosmetic rhinoplasty
**11**	28	M	Severely traumatic deviated nose
**12**	18	F	Severely traumatic deviated nose
**13**	32	F	Cleft lip nasal deformity
**14**	21	M	Cleft lip nasal deformity
**15**	24	F	Secondary to a failing primary cosmetic rhinoplasty
**16**	25	M	Severely traumatic deviated nose
**17**	24	M	Severely traumatic deviated nose
**18**	18	F	Cleft lip nasal deformity
**19**	31	F	Secondary to a failing primary cosmetic rhinoplasty
**20**	20	M	Cleft lip nasal deformity
**21**	23	M	Cleft lip nasal deformity
**22**	21	F	Cleft lip nasal deformity
**23**	38	M	Severely traumatic deviated nose
**24**	42	F	Saddle nose
**25**	21	M	Cleft lip nasal deformity
**26**	24	F	Cleft lip nasal deformity
**27**	21	M	Severely traumatic deviated nose
**28**	35	M	Saddle nose
**29**	31	F	Severely traumatic deviated nose
**30**	19	F	Cleft lip nasal deformity
**31**	28	M	Secondary to a failing primary cosmetic rhinoplasty
**32**	28	M	Secondary to a failing primary cosmetic rhinoplasty
**33**	30	M	Cleft lip nasal deformity
**34**	26	M	Severely traumatic deviated nose
**35**	19	F	Severely traumatic deviated nose
**36**	23	F	Saddle nose

**Table 2:** The Vancouver Scar Scale.

The Vancouver Scar Scale	
Vascularity	
Normal	0
Pink	1
Red	2
**Pigmentation**	
Normal	0
Hypopigmentation	1
Hyperpigmentation	2
**Pliability**	
Normal	0
Supple	1
Yelding	2
Firm	3
Ropes	4
Contracture	5
**Height**	
Flat	0
<2 cm	1
2–5 cm	2
>5 cm	3

**Table 3:** The results of the Vancouver Scar Scale.

Patient	VSS Total Score First Follow-Up Visit *	VSS Total Score Last Follow-Up Visit **
1	2	1
2	3	2
3	1	1
4	2	2
5	2	1
6	1	1
7	2	1
8	1	1
9	1	1
10	2	2
11	2	1
12	1	1
13	1	1
14	1	2
15	2	1
16	1	1
17	3	1
18	2	2
19	1	1
20	1	1
21	2	1
22	1	2
23	2	2
24	1	1
25	1	1
26	1	1
27	2	2
28	2	1
29	1	1
30	2	1
31	1	2
32	1	1
33	1	1
34	3	2
35	1	1
36	1	1
**Average**	**1.53 SD ± 0.64**	**1.28 SD ± 0.45**

* First follow-up visit was at 1 week after surgical treatment. ** Last follow-up visit was 6 months after the surgical treatment.

## Data Availability

Not applicable.
